# *MILK*: a Python scripting interface to *MAUD* for automation of Rietveld analysis

**DOI:** 10.1107/S1600576723005472

**Published:** 2023-07-20

**Authors:** Daniel J. Savage, Luca Lutterotti, Christopher M. Biwer, Michael McKerns, Cynthia Bolme, Marko Knezevic, Sven C. Vogel

**Affiliations:** a Los Alamos National Laboratory, Los Alamos, NM 87545, USA; b Department of Materials Engineering and Industrial Technologies, University of Trento, Trento 38123, Italy; c Department of Mechanical Engineering, University of New Hampshire, Durham, NH 03824, USA; Ecole National Supérieure des Mines, Saint-Etienne, France

**Keywords:** *MILK*, *MAUD*, Rietveld refinement, diffraction software

## Abstract

Modern diffraction experiments (*e.g. in situ* parametric studies) present scientists with many diffraction patterns to analyze. Interactive analyses via graphical user interfaces tend to slow down obtaining quantitative results such as lattice parameters and phase fractions. Furthermore, Rietveld refinement strategies (*i.e.* the parameter turn-on-off sequences) tend to be instrument specific or even specific to a given dataset, such that selection of strategies can become a bottleneck for efficient data analysis. Managing multi-histogram datasets such as from multi-bank neutron diffractometers or caked 2D synchrotron data presents additional challenges due to the large number of histogram-specific parameters. To overcome these challenges in the Rietveld software *Material Analysis Using Diffraction* (*MAUD*), the *MAUD Interface Language Kit* (*MILK*) is developed along with an updated text batch interface for *MAUD*. The open-source software *MILK* is computer-platform independent and is packaged as a Python library that interfaces with *MAUD*. Using *MILK*, model selection (*e.g.* various texture or peak-broadening models), Rietveld parameter manipulation and distributed parallel batch computing can be performed through a high-level Python interface. A high-level interface enables analysis workflows to be easily programmed, shared and applied to large datasets, and external tools to be integrated with *MAUD*. Through modification to the *MAUD* batch interface, plot and data exports have been improved. The resulting hierarchical folders from Rietveld refinements with *MILK* are compatible with *Cinema: Debye–Scherrer*, a tool for visualizing and inspecting the results of multi-parameter analyses of large quantities of diffraction data. In this manuscript, the combined Python scripting and visualization capability of *MILK* is demonstrated with a quantitative texture and phase analysis of data collected at the HIPPO neutron diffractometer.

## Abstract 2

The *MAUD Interface Language Kit* (*MILK*) provides a Python interface to the Rietveld software *MAUD*, enabling scriptable refinements. The *MILK* framework includes parallel computing and folder management which enable high-throughput Rietveld analysis, visualization and validation.

## 1. Introduction

Large datasets from diffraction experiments are routinely collected at synchrotron, X-ray free-electron laser, neutron and other sources to develop a better understanding of materials. The sizes of the datasets collected are expected to continue to grow with detector technology, beamline advancements and science-driven needs to observe sub-second phenomena such as solidification of melt pools during additive manufacturing (Ioannidou *et al.*, 2022 ▸) or self-propagating high-temperature synthesis of MAX phases (Riley *et al.*, 2002 ▸). Diffraction pattern collection rates greater than 1 kHz are currently possible. Even for relatively long count times from neutron diffraction instruments such as the High Pressure/Preferred Orientation (HIPPO) neutron time-of-flight (TOF) diffractometer at LANSCE (Wenk *et al.*, 2003 ▸; Vogel *et al.*, 2004 ▸), a single beam cycle of six months results in tens of thousands of diffraction patterns. Rietveld analysis of HIPPO data is typically one to two orders of magnitude more computationally expensive than analysis of fully integrated synchrotron diffraction data due to the 45 diffraction vectors, multiple sample rotations and large *d*-spacing range (0.3–20 Å) which are fitted (Wenk *et al.*, 2010 ▸). Relatively low quantities of data can thus result in a large analysis burden due to the analysis cost. Therefore, it is desirable to leverage high-performance computing to enable near real time analysis capable of informing or controlling experiments at beamlines.

The processing of large datasets and the subsequent inspection of results does not lend itself to one-off GUI analyses, and has been the motivation for development of several recent diffraction analysis software packages which deal with these common analysis barriers (Clausen, 2003 ▸; Barr *et al.*, 2009 ▸; Baumes *et al.*, 2009 ▸; Vogel, 2011 ▸; Barty *et al.*, 2014 ▸; Ashiotis *et al.*, 2015 ▸; Prescher & Prakapenka, 2015 ▸; Hernández-Rivera *et al.*, 2017 ▸; Vogel *et al.*, 2018 ▸; Orban *et al.*, 2020 ▸; Aimi & Fujimoto, 2020 ▸). None of these software tools are truly universal, and they generally address a single component of the diffraction analysis workflow such as data reduction or data visualization. However, there has been a trend towards development in Python – the current most popular programming language (Carbonnelle, 2022 ▸) – which facilitates the integration of multiple software packages. For example, azimuthal integration is performed using *pyFAI* (Ashiotis *et al.*, 2015 ▸), Rietveld analysis is carried out using the Python interface to *GSAS-II* (Toby & Von Dreele, 2013 ▸) and results are formatted with *pandas* (McKinney, 2011 ▸) for visualization in *Cinema: Debye–Scherrer* (Vogel *et al.*, 2018 ▸).

The advantages of scripting complex analysis workflows become apparent from the nuances of calibrations and analyses. Consider the calibration of multi-histogram neutron texture instruments, for example, HIPPO, GEM or iMATERIA with 45, 328 (Kockelmann *et al.*, 2006 ▸) and 36 (Onuki *et al.*, 2016 ▸) detector panels, respectively. Each histogram requires its own set of calibration parameters for *e.g.* sample position or incident intensity. These beamlines utilize several sample environments (cryostats, furnaces, sample changers *etc.*) that also require their own calibration of sample position and incident intensity. Scripting interfaces greatly simplify the calibration of each detector panel. Communicating the appropriate initialization of a Rietveld refinement, which models to employ and the sequence of parameter fits that are reasonable results in a steep learning curve that can be reduced through scripting. Scripting also creates the possibility of beginning-to-end solutions to data analysis coming from beamlines. Minimal input to scripting frameworks, such as expected phases and runs defining a dataset, can enable automation of calibration, data preprocessing, data analysis, and creating a database for easy exploration and visualization of key quantities.

Quantitative analysis through full pattern fitting using the Rietveld methodology (Rietveld, 1969 ▸) has been widely used in the diffraction community with more than 233 000 publications mentioning or utilizing the Rietveld methodology since 1990 (https://www.dimensions.ai). Rietveld analyses can be performed with open-source software such as *GSAS-II* (Toby & Von Dreele, 2013 ▸), *FullProf* (Rodríguez-Carvajal, 2001 ▸) and *MAUD* (Lutterotti *et al.*, 1999 ▸), or with commercial software such as *JADE* (Jennings, 2021 ▸) and *TOPAS* (Coelho, 2018 ▸). Automation of Rietveld analysis for most software packages either comes with the software, like the scripting language of *TOPAS* (Coelho, 2018 ▸), or requires additional packages such as *gsaslanguage* for *GSAS* (Vogel, 2011 ▸), the Python interface for *GSAS-II* (Toby & Von Dreele, 2013 ▸; O’Donnell *et al.*, 2018 ▸), *srRietveld* for *GSAS* (Tian *et al.*, 2013 ▸) or *autoFP* for *FullProf* (Cui *et al.*, 2015 ▸). We note that sequential refinements, wherein the same set of parameters is varied after simply changing the data that are fitted, is different from script refinements which allow parameter turn-off-on sequences, changing parameter values or models, and conditional refinements. Among the Rietveld programs mentioned, *MAUD* is known for both advanced crystallographic and microstructure models, allowing combined crystal structure, texture, size, strain and stress analyses (Lutterotti, 2010 ▸). In addition, *MAUD* has sample models for part-specific absorption corrections (Xie *et al.*, 2004 ▸; Volz *et al.*, 2006 ▸) and detector models that allow geometrical parameters to be refined with the diffraction data for many neutron and X-ray instruments (Wenk *et al.*, 2010 ▸; Lutterotti *et al.*, 2014 ▸). *MAUD* (Lutterotti, 2023 ▸) is written in Java, and has a variety of detector, X-ray and neutron diffraction analysis capabilities. To date, the means of interacting with the program outside of the GUI has been through the *MAUD* batch mode (Lutterotti, 2005 ▸) – a text-file-based protocol for serialized batch refinements with limited functionality. A simple text-based interface has been identified as a bottleneck in previous work (Raue, 2014 ▸); however, a general scripting interface to *MAUD* has not been developed.

To address the problems identified above, the Python software *MAUD Interface Language Kit* (*MILK*) was developed to enable high-throughput, flexible, scripted *MAUD* analyses. The architecture and philosophy of *MILK* are presented here. To demonstrate the utility of *MILK*, an example is given for a combined quantitative phase and texture analysis of duplex steels from neutron diffraction. The remainder of the paper is structured as follows: in Section 2 the relevant *MAUD* interfaces utilized by *MILK* are described; in Section 3 a description of *MILK* is given; in Section 4 the utility of *MILK* is shown with an example workflow and visualization using *Cinema: Debye–Scherrer*; in Section 5 we discuss on-going work, scalability and fully automating Rietveld analysis at beamlines on the basis of the present stage of *MILK*’s development; and we conclude in Section 6.

## 2. The *MAUD* interface

*MAUD* is designed primarily for GUI refinements, where one can save and load analyses. An analysis is stored on disk as a human readable parameter file (extension .par) following the CIF standard (Hall *et al.*, 1991 ▸) which contains details of the minimization scheme, detector parameters, data and phase objects (among others). The parameter file, in combination with the *MAUD* preference file, is sufficient to store the state of a Rietveld refinement and resume an analysis. Objects in the parameter file are used to compartmentalize parameters and models. Thus, a phase object will not only have properties such as lattice parameters and symmetry, but will also contain sub-objects (*e.g.* models for texture, strain and broadening) which contain their own parameters. *MILK* manipulates the parameter file and calls *MAUD* through the batch interface to execute a refinement and create an updated parameter file. With *MILK*, *MAUD* models can be removed or added; data can be replaced; and parameters can be freed, fixed, constrained through equations, changed or queried. This is accomplished with *MILK* by parsing the *MAUD* parameter file, appropriately editing it and writing it back to disk. A direct and more complicated application programming interface (API) between Python and Java is therefore not needed to script *MAUD* analyses. The parameter names required to interact with the parameter file using *MILK* are available through the *MAUD* parameter tree stored in these *MAUD* parameter files and are available in the *MAUD* GUI parameter list. Names and values of non-refinable parameters, such as the weighting scheme, can be found by viewing the parameter file directly using a text editor. Although some knowledge of the parameter file is needed for the interface to be usable, many of the details are abstracted through a well designed and documented programming interface. Additional information can be found in tutorials and documentation available on the *MILK* website (Savage *et al.*, 2023 ▸).

A batch mode is available through the *MAUD* GUI task bar under ‘Special > Refine in batch…’ in which a user selects a text file that specifies refinement options and input/output parameter files. The current batch interface is similar to that described by Lutterotti (2005 ▸); command options for plotting, texture, and data import and export have been recently extended but are mostly not documented. In addition, batch refinements can be initialized from a command line using the *MaudText* Java routine and specifying a text file with the batch analysis arguments. Rather than supporting operations in *MILK* such as importing data and reading phase CIFs by manipulating parameter files, these operations are handled through the *MAUD* text batch mode. Furthermore, there is some *MAUD* functionality, such as exporting raw fit profiles or plotting texture pole figures, which is not accessible without dedicated *MAUD* routines that are available through the text batch mode. A benefit of off-loading these operations from *MILK* into *MAUD* is a reduction in code maintenance for *MILK* in the event of *MAUD* future releases. Additionally, the *MAUD* analysis wizards are available in the GUI or through the batch interface, and provide several non-extensible, boiler plate analysis templates. The source code for the *MAUD* batch mode interface can be found in the batchProcess.java routine in the source code of *MAUD* (Lutterotti, 2023 ▸), and a detailed description of the interface is provided on the *MILK* GitHub wiki (Savage *et al.*, 2023 ▸).

## 3. The *MILK* framework

*MILK* and its dependencies are configured for installation with minimal user interaction via an *Anaconda* (Anaconda Software Distribution, https://docs.anaconda.com) environment script (see *MILK* wiki), which handles platform-dependent libraries. *MAUD* is under active development and is available at http://maud.radiographema.eu compiled for Windows, Linux, MacOS/Intel and MacOS/ARM platforms. For full functionality, the latest version of *MAUD* is recommended (current version at the time of writing is 2.998). *MILK* interfaces with *Cinema* and *MAUD* using environment variables that specify their respective installation paths. The *MILK* module can then be imported in a Python session, and all the *MAUD* functionality (pair distribution function, X-ray and neutron diffraction, X-ray reflectometry, and X-ray fluorescence) is available through standard object-oriented programming in which classes are initialized and interfaces prompted for the necessary input when using an integrated development environment (IDE) such as Visual Studio Code or Spyder. There are two classes which perform the core functions of *MILK*, the *editor* class, providing an interface to *MAUD* parameter files, and the *maudText* class, encapsulating access to the *MAUD* text interface. Using the *config* method of each class, *editor* and *maudText* parameters are initialized with values from a project-specific milk.json file (*e.g.* paths to the *MAUD* executable, filenames for intermediate files *etc.*), but parameters can be overwritten in a *MILK* Python script as needed.

The high-level *editor* class operates on a *MAUD* parameter file. Currently, the *editor* class has the core methods listed in Table 1 ▸. In addition, the class has support for changing the texture model and size–strain model as well as their parameters. Other *MAUD* models that are not supported by *MILK* [*e.g.* strain models (Wenk *et al.*, 2014 ▸)] can be added to a *MILK* analysis by including the models in a template file used to initialize an analysis. It is the intention of the *MAUD* and *MILK* developers that all models will eventually be available through the *MAUD* text interface, since this ensures complete compatibility and proper integration. The methods of the *editor* class are documented in the *MILK* wiki (Savage *et al.*, 2023 ▸) and have method documentation in the Python environment. Collectively, these methods allow parameters to be fixed, refined, queried and tracked in output files, and constrained together; background models in *MAUD* to be manipulated (*e.g.* changing the number of polynomial coefficients overall or per histogram); the state of the parameter file to be summarized (*e.g.* how many parameters are varied); and texture models to be manipulated (see Table 1 ▸ for an overview of the methods).

*MILK* utilizes a simple interface to control the scope of changes to a parameter file. To target a specific parameter, a string of text, for example, cell_length_a is assigned to the *key* argument. The parameter file is searched for that key. Several matches may occur (*e.g.* if there are two phases then there will be two sets of lattice parameters with the same key cell_length_a, or if the search is for cell_length then cell_length_a, cell_length_b and cell_length_c will be found), and to further limit the scope a list of *sobj* (subordinate objects to include, *e.g.* the name of a phase) or *nsobj* (subordinate objects to exclude) can be specified. The ability to filter subordinate objects is sufficient to control the scope of an *editor* method given the object structure in the *MAUD* parameter file. If the targeted *key* is a loop variable, such as a list of atoms or background parameters, a *loopid* can be specified to further limit the scope of the method to a single variable in the loop.

To work efficiently over many Rietveld analyses and store results, *MILK* utilizes a directory structure wherein each analysis is contained in its own folder and each refinement step is archived in a subfolder. *MILK* operates over a list of paths and provides means to generate a subfolder structure to organize analysis of large datasets. With this folder structure, modifications to many parameter files can be performed from a single *editor* method call. *MILK* can also operate on a subset of the list of paths, thereby limiting the scope of changes or refinements across analysis folders (*e.g.* to test an analysis on a subset of runs before analyzing the entire project). See the *MILK* documentation for more details (Savage *et al.*, 2023 ▸).

Complementing the *editor* class is the *maudText* class, which is a high-level interface for encapsulating writing *MAUD* textmode input files, calling *MAUD* from the command line to process the input files and storing outputs. The *refinement* method of *maudText* performs a refinement based on the arguments of the method, such as the number of iterations, and from the milk.json initialization, such as pole figures to be produced as PNG files. There are runtime Boolean options when calling *refinement* that control plotting, phase import, data import *etc.* *MILK* replaces CIF-format-specific knowledge needed to utilize the *MAUD* text interface with a more intuitive Python interface and preconfigured options for plotting and data output. Each time the *refinement* method is called a new step is created which is associated with that refinement, allowing a sequence of refinements to be saved by step number. Because of the *MILK* folder structure, the Python multiprocess is utilized to run refinements in parallel without interference. The *MAUD* console and error messaging are streamed to .log and .err extension files, respectively, in each analysis folder, allowing inspection and debugging of the *MAUD* runtime output. Full details of the options are provided in the *MILK* documentation (Savage *et al.*, 2023 ▸).

The outputs of the Rietveld analysis, *MAUD* parameter files, graphics and text files with tracked parameters are saved in each analysis folder and archived according to the step number to a subfolder. The outputs allow different levels and types of inspection to be performed, ranging from text file parameter inspection, to inspecting texture and 2D histogram fits, to opening a parameter file in the *MAUD* GUI. The folder convention, including the archived steps, is compatible with the *Cinema* database convention, enabling seamless integration of *Cinema: Debye–Scherrer* (Vogel *et al.*, 2018 ▸) for inspection and visualization. *Cinema* can be used not only for inspection, filtering and exploration of the final results but also to efficiently identify problems in multistep Rietveld analyses of large datasets.

In parametric studies, the starting values for a lattice parameter (*e.g.* as a function of temperature) or phase fraction (*e.g.* due to phase transformations) often need to be modified to ensure convergence of Rietveld refinements. To facilitate efficient analysis of large datasets, *MILK* generates a dataset.csv file that can be manually edited using spreadsheet software to include metadata (*e.g.* sample temperature) and handle dissimilar diffraction data by adjusting parameter starting values (*e.g.* lattice parameters or phase fractions). The metadata can easily be incorporated into the *Cinema* visualization to plot, for example, lattice parameter versus temperature. Use of dataset.csv for this purpose is illustrated below, and a more complex example is provided on the *MILK* wiki (Savage *et al.*, 2023 ▸).

## 4. Example workflow: HIPPO quantitative phase and texture analysis

To illustrate the analysis automation and result inspection enabled by *MILK* and *Cinema*, a typical application case of texture and phase fraction analysis with setup and data initialization for the HIPPO diffractometer is presented in this section. Though specifics of the neutron TOF data analysis are discussed, the workflow for other diffraction data analyses is similar. The scripted procedure has been robust in processing several hundred HIPPO diffraction datasets. In addition, *MILK* has been applied extensively on synchrotron X-ray and X-ray free-electron laser datasets (Savage *et al.*, 2023 ▸), and example scripts for these analysis types are provided on the *MILK* GitHub wiki. The code and data for the example discussed in this section are included with *MILK* and can be copied to a working directory by importing *MILK* into a Python environment and subsequently calling MILK.examples.hippo_texture(). As unique *MILK* applications grow and datasets are released by collaborators, example scripts will be added to the *MILK* GitHub site and documented.

### 4.1. Setup and inputs

**Figure 1:**
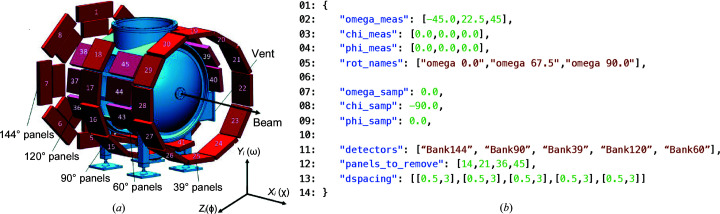
(*a*) Schematic of the HIPPO diffractometer at LANSCE. (*b*) Input hippo.json used to define the set of measurement rotations and sample rotations and for the definition of the detector panels, banks per ring, banks to exclude and *d*-spacing range for each of the detector panels. This input parameter file is parsed in the setup.py script, allowing a simple interface to configure HIPPO and experiment-specific parameters.

This example is included with *MILK* as a template for quantitative phase and texture analysis with the HIPPO TOF neutron diffractometer. Phase and texture analysis for other beamlines will require a different setup script, but the subsequent refinement procedures should work with only minor adjustment once the detector and data are configured for a texture analysis. The detector configuration for HIPPO has been the same since 2012 and consists of 45 panels arranged on 5 detector rings at 2θ = 144, 120, 90, 60 and 40°, respectively (Takajo & Vogel, 2018 ▸). See Fig. 1 ▸(*a*) for the HIPPO detector layout. Since the 2012 upgrade, three sample rotations are typically used for texture analysis on HIPPO. The analysis therefore includes simultaneous fits to 135 histograms, similar to analysis of caked azimuthal integration and sample rotations in synchrotron texture measurements (Lutterotti *et al.*, 2014 ▸). The number of histograms makes it non-trivial to set up and manage (vary, fix, change value *etc.*) hundreds of histogram-specific parameters (*e.g.* scale factors, backgrounds, peak profile parameters and TOF to *d*-spacing conversion factors). Setup and analysis were hitherto carried out in *MAUD* using the predefined HIPPO import and general analysis wizards. *MILK* offers the same functionality, but with increased flexibility for data import, analysis and automation.

As discussed above, the *editor* class modifies existing parameter files; therefore, a template *MAUD* parameter file (.par) is modified to configure a new analysis. Best practice is to make a template analysis file for a given instrument configuration with the user’s version of the *MAUD* GUI, where detector objects (*e.g.* from multiple rotations) and functions (*e.g.* peak profile functions and absorption models) are specified, since data layouts occasionally change. In general, *MAUD* is backwards compatible with previous releases, but there may be preference options that need to be changed for complete compatibility. For completeness of the *MILK* examples, a template parameter file from the current *MAUD* release (version 2.998) is provided.

Three input files are used to modify the *MAUD* template parameter file for HIPPO datasets: (i) milk.json is used for every *MILK* analysis and is a file used to initialize the *editor* and *maudText* instances (*e.g.* path to *MAUD* installation, directory names and phases to import); (ii) dataset.json is used to specify *MILK* information such as the relative path to the data, the data grouping (*e.g.* three *GSAS*-format gda files representing three rotations belonging to one analysis) and the filename of the aforementioned template parameter file for the analysis; (iii) lastly, hippo.json [see Fig. 1 ▸(*b*)] is used to specify HIPPO-specific metadata, allowing general analysis pipelines to be defined (*e.g.* excluded detector panels and arbitrary rotation angles). The use of these JavaScript Object Notation (JSON) files provides a defined interface for users of the *MILK* analysis script and removes much of the otherwise required modification of these analysis scripts.

For complicated neutron TOF instrument configurations, *MAUD* utilizes a *GSAS*-format instrument parameter file (iparm) to define the detector geometry, peak profile parameters *etc*. In Fig. 1 ▸(*a*) we show a schematic of the HIPPO detector layout, and in Fig. 1 ▸(*b*) the corresponding parameters needed to configure a *MILK* analysis with HIPPO are defined. The number of each panel overlaid on Fig. 1 ▸(*a*) corresponds to the banks and banks_remove parameters in the hippo.json. The six-axis HIPPO robotic sample changer (Losko *et al.*, 2014 ▸) was used to create the example dataset with omega rotations at 0.0, 67.5 and 90.0° and chi and phi of 0.0 for each measurement orientation. For this reason a list of three lengths for the omega_meas, chi_meas, phi_meas and rot_names variables is used. Note that the template has rotation names such omega 67.5 which are specific to when the template was made with the HIPPO texture wizard, and these strings are stored in the template; however, using the search and replace functionality of the *MILK* *editor* class, the omega_meas, chi_meas and phi_meas values can be set to the actual values used during a measurement. In the example experiment, the robot omega rotation angles −45.0, 22.5 and 45.0° were used, instead of the 0.0, 67.5 and 90° rotation angles of the template file. rot_names specifies the string to append to the Bank### detector name and creates the string to filter subordinate objects in the parameter file. In addition to the definition of the sample rotations during the measurement, three rotations omega_samp, chi_samp and phi_samp can be specified that rotate the sample frame in *MAUD* in which sample-specific models, such as texture, are defined. The −90.0° rotation chi_samp brings the omega rotation axis into the center of *MAUD* pole figures for HIPPO datasets (Wenk *et al.*, 2010 ▸). The bank panels to exclude (*e.g.* because the panel is shielded by the sample environment) and the *d*-spacing range for each detector ring can also be modified. While the detectors definition should not be modified for texture wizard template files unless the instrument parameter file is modified, it is provided in hippo.json to define the sequence of dspacing ranges to be the same as the sequence of detectors. This set of strings is also used to manipulate bank-specific parameters such as histogram scale factors and diffractometer constants such as DIFC in *MAUD* multi-bank detector definition. The concept of the input JSON files can be adapted to any beamline- or data-analysis-specific needs to create highly flexible *MILK* analysis workflows that require limited modification of the *MILK* scripts.

### 4.2. The analysis pipeline

**Figure 2:**
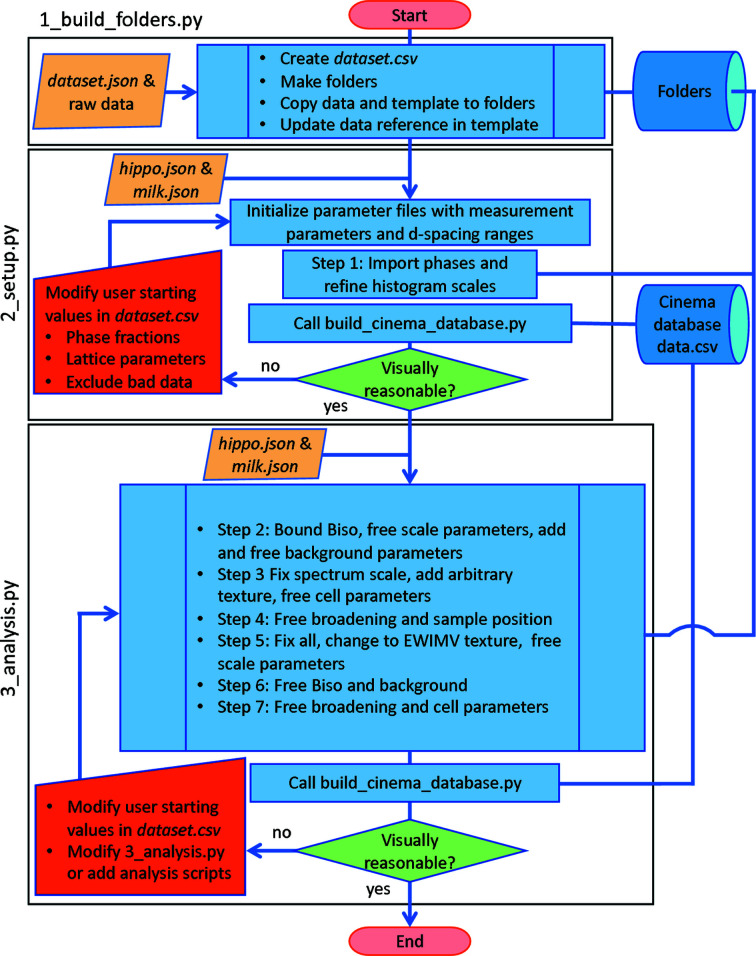
Analysis workflow for the three Python files performing key tasks during an example *MILK* refinement. 1_build_folders.py groups diffraction data into folders, 2_setup.py initializes the Rietveld to a good starting point and 3_analysis.py performs a sequence of refinement steps that result in a high-quality fit. At the end of the last two Python files, the *Cinema* data.csv file is created to visualize the results with *Cinema: Debye–Scherrer*.

The *MILK* analysis pipeline from folder creation to final refinement is outlined in Fig. 2 ▸. The analysis is separated into three *MILK* scripts that, once validated, do not need to be repeated (1_build_folders.py, 2_setup.py and 3_analysis.py to be executed in this sequence). HIPPO diffraction data are grouped and copied into folders in 1_build_folders.py such that each measurement on a sample is in its own folder (*i.e.* the three datafiles from the three omega rotations). HIPPO data are imported into a *MAUD* parameter file by swapping the dataset names in the file and removing any stored diffraction data in the file. When the parameter file is next loaded into *MAUD*, the referenced diffraction data will automatically be imported. dataset.csv, introduced earlier in Section 3, is created by 1_build_folders.py to facilitate setting of starting values, excluding runs, providing metadata *etc*. Initialization of key Rietveld parameters and experimental parameters from hippo.json is performed in 2_setup.py. Visualization using Cinema of the diffractograms at the end of this Python script allows verification of a reasonable starting parameter set (correct phases, appropriate lattice parameters *etc*.) and provides the ability to exclude bad datasets. User corrections of starting values and exclusion of bad runs can be accomplished by editing dataset.csv using a spreadsheet program, followed by rerunning 2_setup.py. Building on the example functions in 2_setup.py, users can add columns in dataset.csv to enable, for example, prescribing temperature-dependent atomic displacement parameters for a heating study to obtain more reliable phase fractions from the Rietveld analysis. The Rietveld refinements leading to the final result are performed in 3_analysis.py. The refinement strategy in this example consists of two components: (i) using an arbitrary texture model (a Le Bail fit) to fit lattice parameters and peak broadening, which provides a very good fit of background and peak profiles; and (ii) refining a texture model while sequentially freeing parameters as the refinement progresses.

**Figure 3:**
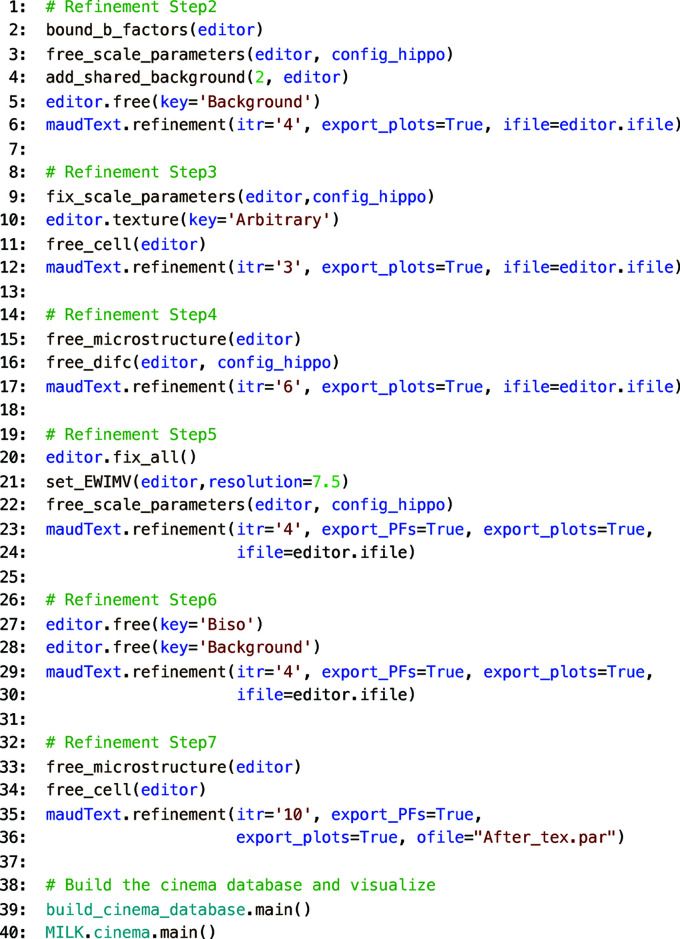
Python code from 3_analysis.py used to run the sequence of refinements described in Fig. 2 ▸, build a *Cinema* database, configure the *Cinema* inputs and launch a *Cinema* instance in the default web browser.

The sequential Rietveld analysis strategy in 3_analysis.py has proven robust unless there are low volume fraction phases, many phases or significant peak overlap of phases. There are likely to be many scenarios in which modifying the 3_analysis.py script will be required. Fig. 3 ▸ shows a code example, demonstrating the sequence of refinements outlined in Fig. 2 ▸. Rearranging the sequence of parameter turn-on-off in 3_analysis.py allows different, more complicated Rietveld analysis strategies to be implemented using the high-level *MILK* interface to *MAUD*. The HIPPO analysis pipeline for quantitative phase and texture analysis described here is available with the *MILK* installation and can be used as starting scripts for other applications.

**Figure 4:**
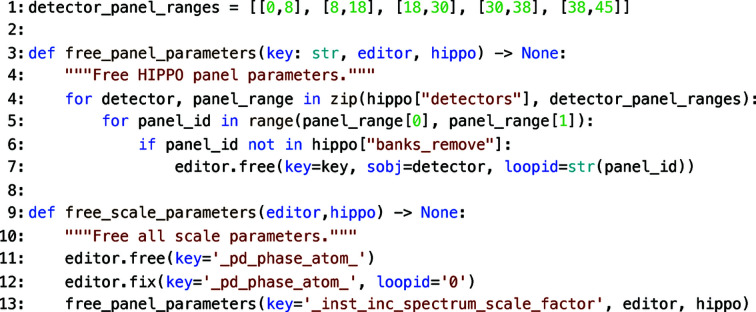
Example Python code from 3_analysis.py used to free scale parameters and demonstrate how the HIPPO-detector-specific parameters are looped through. Using instrument-specific functions like free_scale_parameters and using collections of lower-level functional calls such as the function free_scale_parameters ensure the sequence of refinements remains modular without loss of functionality.

In Fig. 3 ▸ there are functions (*e.g.* free_scale_parameters) that are defined to encapsulate several *MILK* commands or instrument-specific parameter handling, thereby keeping a sequence of refinements modular and high level. An example of the free_scale_parameters function from 3_analysis.py is given in Fig. 4 ▸ to illustrate how HIPPO scale factors are freed using *MILK*. Included in the scale parameters is the phase atomic fractions which are stored in the *MAUD* parameter file as a loop. The sum of the phase fractions is continuously normalized to 1 during Rietveld refinements. To avoid correlations in scale factors in the case of a multi-detector array instrument such as HIPPO, one phase should have its fraction fixed and, if only one phase is present, the phase fraction should not be refined (see lines 11 and 12 in Fig. 4 ▸). The free_panel_parameters function is a general function that loops through the layout of HIPPO panels as specified in hippo.json and frees the loop parameters associated with a specified key (for example, the _inst_inc_spectrum_scale_factor script in Fig. 4 ▸).

### 4.3. Visualization

The visual inspection of Rietveld refinements is of paramount importance for the data analysis process, more important than simple inspection of scalar goodness-of-fit parameters such as *R*_wp_ (Toby, 2006 ▸). Efficient inspection of multistep Rietveld analysis of large datasets requires special tools, as the *MAUD* GUI is only capable of visualizing one parameter file at a time. The HTML-based *Cinema: Debye–Scherrer* was developed to facilitate visualization of large Rietveld datasets (Vogel *et al.*, 2018 ▸). Prior to *MILK*, *MAUD* was not compatible with *Cinema: Debye–Scherrer*. The *MILK* storage scheme for each Rietveld refinement step allows individual steps to be visualized and evaluated with *Cinema*, thus enabling efficient and complete inspection of the Rietveld analysis. The *Cinema* framework was originally developed for visualization at extreme scales (Ahrens *et al.*, 2014 ▸) and can also be used for real time or *post hoc* visualization of raw diffraction images, a velocity interferometer system for any reflector (VISAR), equations of state and other relevant diffraction experiment information (Woodring *et al.*, 2017 ▸; Orban *et al.*, 2020 ▸; Biwer *et al.*, 2021 ▸).

For construction of the database, the script employed, build_cinema_database.py, finds the parameter files, retrieves parameters of interest, and collects PNG files of 1D and 2D histograms (produced during 2_setup.py and 3_analysis.py and equivalent to plots available through the *MAUD* GUI) into a *Cinema*-compatible database that is saved to data.csv in the root folder of the *MILK* analysis. All PNG files residing in the *MILK* folders are referenced in data.csv.

**Figure 5:**
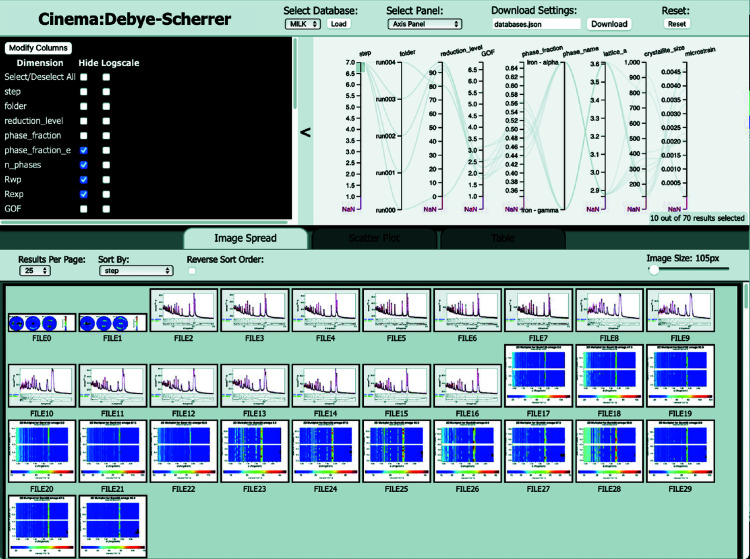
Example of the *Cinema: Debye–Scherrer* dashboard, visualizing the final refinement run of the HIPPO texture and phase *MILK* analysis tutorial. Filter options (upper left) for the parallel plot (upper right) together allow interaction with the Rietveld steps and data. Filters applied on the parallel plot (*e.g.* only step_7 is active) allow the ‘Image Spread’, ‘Scatter Plot’ and ‘Table’ section to be filtered. These features allow large databases of Rietveld analyses to be quickly visualized and problems (*e.g.* missing phases or the step where parameters run away) to be identified.

In Fig. 5 ▸ the HIPPO tutorial dataset produced with 3_analysis.py is visualized with select parallel plot columns enabled (check boxes in upper left corner) and only the last Rietveld step, step_7, is selected (highlighted region on the leftmost vertical axis of the parallel plot). Due to the *MILK* folder structure, which contains each Rietveld step, each step of the Rietveld process can be accessed, and thus any issues that may occur (*e.g.* runaway parameter) can be identified and the scripts modified to address the issue at the step in which they occur. The ‘Image Spread’ tab at the bottom shows the refinement of the first dataset as PNG files of 1D, 2D (of the different detector banks and sample rotations) and pole figure plots which are exported from *MAUD*. A magnified image can be viewed by clicking on an image or by changing the ‘Image Size’. Though not shown, the metadata can be plotted in an *xy* scatter plot (*e.g.* phase fraction versus time or lattice parameter versus temperature) in the ‘Scatter Plot’ tab and viewed in a table format in the ‘Table’ tab. For more information, see the tutorial on the *MILK* wiki (Savage *et al.*, 2023 ▸; Vogel *et al.*, 2018 ▸). By identifying problems with refinement steps or parameters, the 3_analysis.py or input parameters (*e.g.* from dataset.csv) can be modified and the next iteration of the analysis process can be started.

## 5. Ongoing and future developments

The framework described here for scripted Rietveld analysis is distinct from other software design such as the *GSAS-II* Python interface in that parallel computation is applied whenever diffraction data dependencies are not present. The example in Section 4 demonstrated a speed-up proportional to the number of cases so long as the available computer resources (*i.e.* CPUs and memory) do not exceed the demands of the diffraction datasets. The simple parallelism over unconnected data is highly effective at reducing Rietveld analysis time and the time needed to iterate over a refinement strategy. As of *MAUD* version 2.998, *MILK* contains a ∼1 s overhead for every refinement that comes from starting and closing the Java instance. We are currently pursuing the use of persistent Java instances to limit this overhead to a one-time initialization. Such an overhead is not present in *GSAS-II* Python and is mainly important in Rietveld analyses of single diffractograms, which are often less than 1 s in duration and are routinely carried out during real time analysis. Expensive Rietveld analyses like in the Section 4 example take minutes to complete and are less affected by the overhead. Currently we are pursuing using our framework on high-performance computing resources, with the goal of providing near real time diffraction analysis. The data management strategies (*i.e.* the separation of files for an individual analysis into its own folder) and parallel computation developed with *MILK* can be applied with little extension to other Rietveld software if a Python interface is available. Therefore, in the future *GSAS-II*, for example, could be used as the Rietveld analysis tool in the *MILK* parallel framework.

By having a Python interface for the Rietveld process, different schemes can be implemented to identify suitable starting values that lead to convergence of the fit process. This opens pathways to apply machine learning or artificial intelligence based concepts into the Rietveld process. Removing the human trial-and-error approach to Rietveld analysis using advanced optimization and models will allow full Rietveld analysis automation of most datasets. In essence, *MILK* provides the interface between highly specialized, complex diffraction data analysis and powerful tools and libraries for which Python interfaces exist. Examples range from optimizers (McKerns *et al.*, 2012 ▸; Gagin & Levin, 2015 ▸), to state-of-the-art machine learning libraries (Abadi *et al.*, 2016 ▸; Paszke *et al.*, 2019 ▸), to deformation models describing lattice strain and texture evolution under applied load (Ferreri *et al.*, 2022 ▸). In other words, *MILK*, like the Python interface to *GSAS-II*, enables the user community to add functionality to Rietveld analysis applications without relying on the efforts of Rietveld code maintainers. Building on this concept, our group is integrating the Mystic library (McKerns *et al.*, 2012 ▸) to provide uncertainty quantification and surrogate modeling to rapidly identify starting values for Rietveld analysis and certify the optimality of refinements, again with the end goal of providing near real time diffraction data analysis. Improving the integration of *MILK* with *MAUD* by modifying the *MAUD* source code (*e.g.* to improve graphical output, optimize parameter file disk operations *etc*.) is also ongoing.

## 6. Summary

The Python-based *MAUD Interface Language Kit* (*MILK*) introduces a Python interface, parallel computing, data management and compatibility with visualization using the *Cinema* database protocol into Rietveld analysis with *MAUD*. The high-level Python interface of *MILK* enables complex workflows to be implemented and large diffraction datasets to be efficiently analyzed due to its visualization integration and parallel computing capabilities. The software is compatible with Mac OS, Linux and Windows and can be downloaded from https://github.com/lanl/MILK, where detailed program documentation, tutorials and example workflows are also hosted.

Example data and documentation can be found at https://github.com/lanl/MILK.

## Figures and Tables

**Table 1:** List of *editor* class methods and descriptions of the change in the parameter file

Method	Description
free	Set parameter to refined
f ix	Set parameter to not refined
f ix_all	Set all parameters to not refined
set_val	Set value of parameter
get_val	Obtain value of parameter
get_err	Obtain uncertainty of parameter
get_phases	Obtain list of phase names
ref	Constrain one parameter to another parameter through an equation
unref	Remove constraint between parameters
add_dataf ile_bk_par	Add a background parameter to individual spectra
add_loop_par	Add parameter to loop variable (see text)
rem_loop_par	Remove parameter from loop variable
reset_odf	Reset the ODF to a uniform distribution
remove_obj	Remove an object in the parameter file such as a phase or detector
track	Output parameter value to result text file
untrack	Stop output of parameter value to result text file
untrack_all	Stop all output of parameters to the result text file
summary	Print parameter file statics (*e.g.* the number of free variables and which variables are freed)
